# Supplementary material for: Antagonistic Effects of IL-4 on IL-17A-Mediated Enhancement of Epidermal Tight Junction Function
Source: Int J Mol Sci. 2019 Aug 21;20(17):4070. doi: 10.3390/ijms20174070 (PMC6747459; doi:10.3390/ijms20174070)
Supplement: Supplementary file 1 [file ijms-20-04070-s001.pdf]

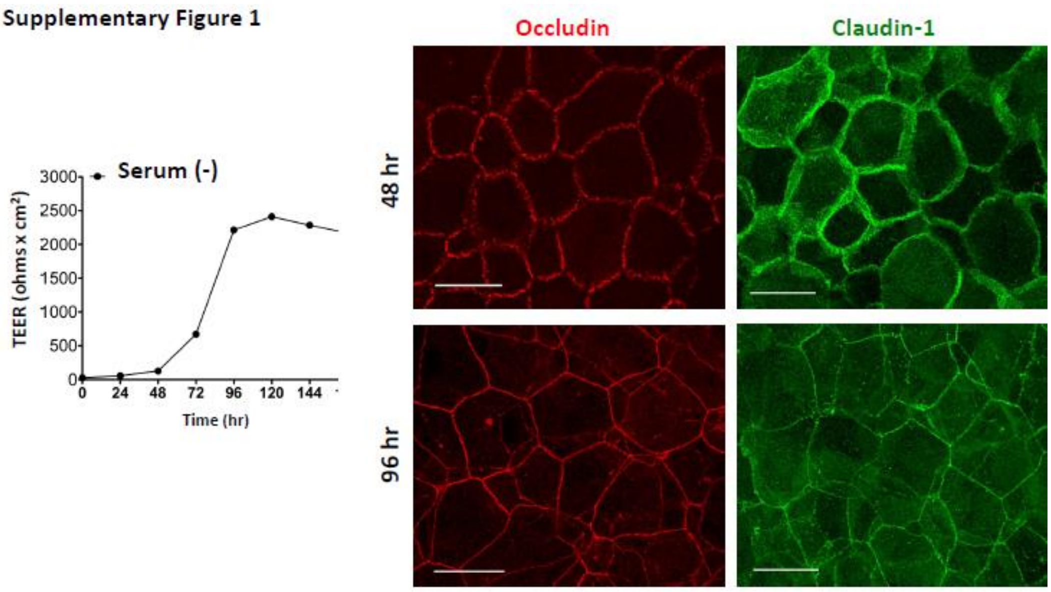

Figure S1. PHK differentiated in high calcium containing media depleted of bovine serum or growth factors (serum -) form a barrier to ion flux, as measured by increased TEER, that peaks around 96 hours post differentiation. Representative immunostaining of Occludin (red) and CLDN1 (green) in PHK cultured in serum (-) at 48 h and 96 h post differentiation showing the maturation of a characteristic TJ "chicken wire" pattern. Scale bar = 40 µm.

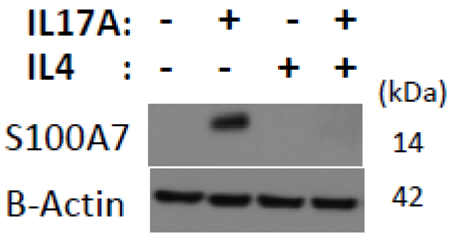

Figure S2. IL-4 blocks IL-17A induced S100A7 expression in PHK. A representative Western blot is shown (n = 3 experiments).

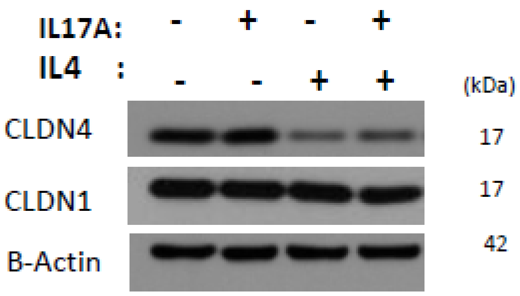

Figure S3. CLDN4 expression is reduced by IL-4 treatment in both media alone and IL-17A treated PHK in the submerged culture model. No changes were observed in CLDN1 expression. A representative western blot is shown.
